# Associations of *collagen type 1 α1* gene polymorphisms and musculoskeletal soft tissue injuries: a meta-analysis with trial sequential analysis

**DOI:** 10.18632/aging.205846

**Published:** 2024-05-22

**Authors:** Rui Guo, Shutao Gao, Nazierhan Shaxika, Aihaiti Aizezi, Haidi Wang, Xiang Feng, Zhigang Wang

**Affiliations:** 1 Department of Orthopedic Center, People’s Hospital of Xinjiang Uygur Autonomous Region, Urumqi, Xinjiang 830001, China; 2 Department of Spine Surgery, Xinjiang Medical University First Affiliated Hospital, Urumqi, Xinjiang 830054, China

**Keywords:** COL1A1, polymorphism, musculoskeletal soft tissue injury, anterior cruciate ligament injury, meta-analysis

## Abstract

Numerous studies have investigated the role of *collagen type 1 α1* (*COL1A1)* polymorphisms in musculoskeletal soft tissue injuries (MSTIs), yielding conflicting results. This study was designed to synthesize existing evidence and clarify the relationship between *COL1A1* polymorphisms and MSTI susceptibility. We conducted a comprehensive literature search using PubMed, Cochrane Library, Web of Science, EMBASE, and Wanfang databases. Associations were assessed using odds ratios (ORs) with 95% confidence intervals (95% CIs) across five genetic models. Subgroup analyses were performed based on ethnicity and injury type. Additionally, trial sequential analysis (TSA) was utilized to assess information size and statistical power. We analyzed a total of 16 articles from 358 retrieved studies, encompassing 2094 MSTI cases and 4105 controls. Our pooled data revealed that individuals with the TT genotype of the *rs1800012* polymorphism had a significantly reduced risk of MSTIs (TT vs. GG, OR = 0.53, 95% CI 0.35–0.82, *P* = 0.004; TT vs. TG + GG, OR = 0.54, 95% CI 0.36–0.80, *P* = 0.002). Ethnicity-based stratification showed a significant association in Caucasians but not Asians. However, no significant association was observed between the *rs1107946* polymorphism and MSTIs, regardless of ethnicity or injury type. TSA indicated that the sample sizes may have been insufficient to yield conclusive results. In conclusion, our study supports the protective effect of the TT genotype of the *rs1800012* polymorphism against MSTIs, particularly among Caucasians. However, the *rs1107946* polymorphism does not appear to influence MSTI susceptibility.

## INTRODUCTION

Musculoskeletal soft tissue injuries (MSTIs), including ligament, tendon, and muscle injuries, commonly occur during recreational or occupational activities, affecting both athletes and non-athletes. It is estimated that 47% of children and adolescents have experienced at least one physical activity-related injury [1]. However, determining the exact incidence of MSTIs in the general population remains challenging. During the 2016 Olympic Games, 8% of athletes sustained sports-related injuries [2], highlighting the significant impact of MSTIs on individuals’ well-being, work capacity, and participation in physical activities, along with the substantial economic burden associated with managing these conditions [3].

The etiology of MSTIs remains largely unclear, with both intrinsic (such as genetic factors) and extrinsic (such as physical activity and chronic overuse) factors being proposed as contributing factors [4, 5]. Inherited genetic factors may predispose individuals to an increased or decreased risk of MSTIs. In recent years, numerous studies have focused on decoding the genetic basis of MSTIs to understand the underlying molecular mechanisms [6]. To date, a multitude of genes have been implicated in MSTIs, including collagen-encoding, transforming growth factor-β (TGF-β), matrix metalloproteinase (MMP), and growth/differentiation factor genes [7].

Collagens, the predominant proteins in mammals, play a crucial role as the major structural proteins in ligaments and tendons. Type I collagen (COL1) accounts for nearly 90% of the total content in ligaments and tendons, consisting of two α1 chains and one α2 chain, providing structural and mechanical stability to biological tissues [8].

Khoschnau et al. [9] initially reported the *rs1800012* polymorphism in the *COL1A1* gene, which was significantly associated with a decreased risk of shoulder dislocation and cruciate ligament rupture. This study sparked further investigation into this area. Several studies have explored the role of the *COL1A1* polymorphisms in MSTI susceptibility [10–19]. However, the findings have been mixed and inconclusive due to variations in ethnic backgrounds, clinical heterogeneity, and gender differences. This study was designed to synthesize the existing evidence to assess the conflicting results and elucidate the correlations between *COL1A1* polymorphisms and susceptibility to MSTIs.

## MATERIALS AND METHODS

### Literature search

A comprehensive literature search was conducted across multiple databases, including Web of Science, PubMed, EMBASE, Cochrane Library, and Wanfang. The keywords for literature search string were: (Achilles tendon OR Tendon injury OR Tendinopathy OR Achilles tendon rupture OR ACL injury OR Anterior cruciate ligament injury OR Ligament injury OR Anterior cruciate ligament tear OR ACL tear OR Tennis elbow OR Lateral epicondylitis OR Rotator cuff tear OR Musculoskeletal injury OR Muscle injury OR Soft tissue injuries OR Tendon-ligament injuries) AND (Collagen Type I Alpha I OR Collagen Type I Alpha1 OR Collagen Type1 Alpha 1 OR Collagen Type I α1 OR Collagen Type 1 α1 OR Type 1 Collagen α1 OR Type I Collagen α1 OR COL1A1) AND (Mutation OR Variant OR Variation OR Polymorphism). Literature search was conducted without any restriction on language. The reference lists of eligible studies were screened for additional relevant articles. Two authors (RG and SG) independently performed the literature search, with any discrepancies resolved by a third author.

### Inclusion and exclusion criteria

Inclusion criteria comprised (i) studies examining the associations between *COL1A1* polymorphisms and MSTIs; (ii) studies in which MSTI diagnosis was established through clinical evaluation, imaging, or surgery; (iii) studies in which healthy individuals without MSTIs served as controls; and (iv) studies in which detailed genotype data were available to calculate the odds ratios (ORs) and 95% confidence intervals (95% CIs). Exclusion criteria included (i) duplicate publications; (ii) reviews, conference abstracts, commentary articles, or case reports; and (iii) animal studies. In cases of overlapping data, only the most comprehensive study was included.

### Evaluation of study quality

Two authors (RG and SG) independently assessed study quality using the Newcastle-Ottawa Scale (NOS), considering “selection,” “comparability,” and “outcome” criteria, with discrepancies resolved by consulting a third investigator. Studies scoring >5 points were deemed high quality.

### Data extraction

Two review authors (RG and SG) independently extracted relevant information, including author details, publication year, country of origin, ethnicity, gender, study type, diagnostic methods, genotyping techniques, genotype counts, and Hardy-Weinberg equilibrium (HWE) test results [20]. Discrepancies were resolved by a third investigator.

### Statistical analysis

Associations were assessed using ORs and 95% CIs. The pooled effect size was calculated under the allele (T vs. G), homozygote (TT vs. GG), heterozygote (TG vs. GG), dominant (TT + TG vs. GG), and recessive (TT vs. TG + GG) models. Heterogeneity was evaluated using Q-statistics and I^2^-statistics, with data pooled using fixed-effect or random-effect models based on heterogeneity levels (*P* > 0.10, I^2^ ≤ 50%). HWE in the control group was assessed using the chi-squared test. Subgroup analyses were conducted based on ethnicity and injury type. Statistical analyses were performed using RevMan 5.3 software.

### Sensitivity analysis and publication bias

To assess the reliability and robustness of the pooled outcomes, we conducted a sensitivity analysis by sequentially excluding each study and recalculating the ORs and 95% CIs. Additionally, we utilized funnel plots and Egger’s and Begg’s linear regression tests to examine potential publication bias.

### Trial sequential analysis

We performed trial sequential analysis (TSA) to estimate the required information size (RIS) based on a 20% relative risk reduction, a 5% overall type I error, and an 80% statistical test power [21]. TSA was conducted using TSA 0.9.5.10 Beta software.

### Availability of data and materials

All data generated during this study are included in this published article.

## RESULTS

### Literature identification

**Figure 1:**
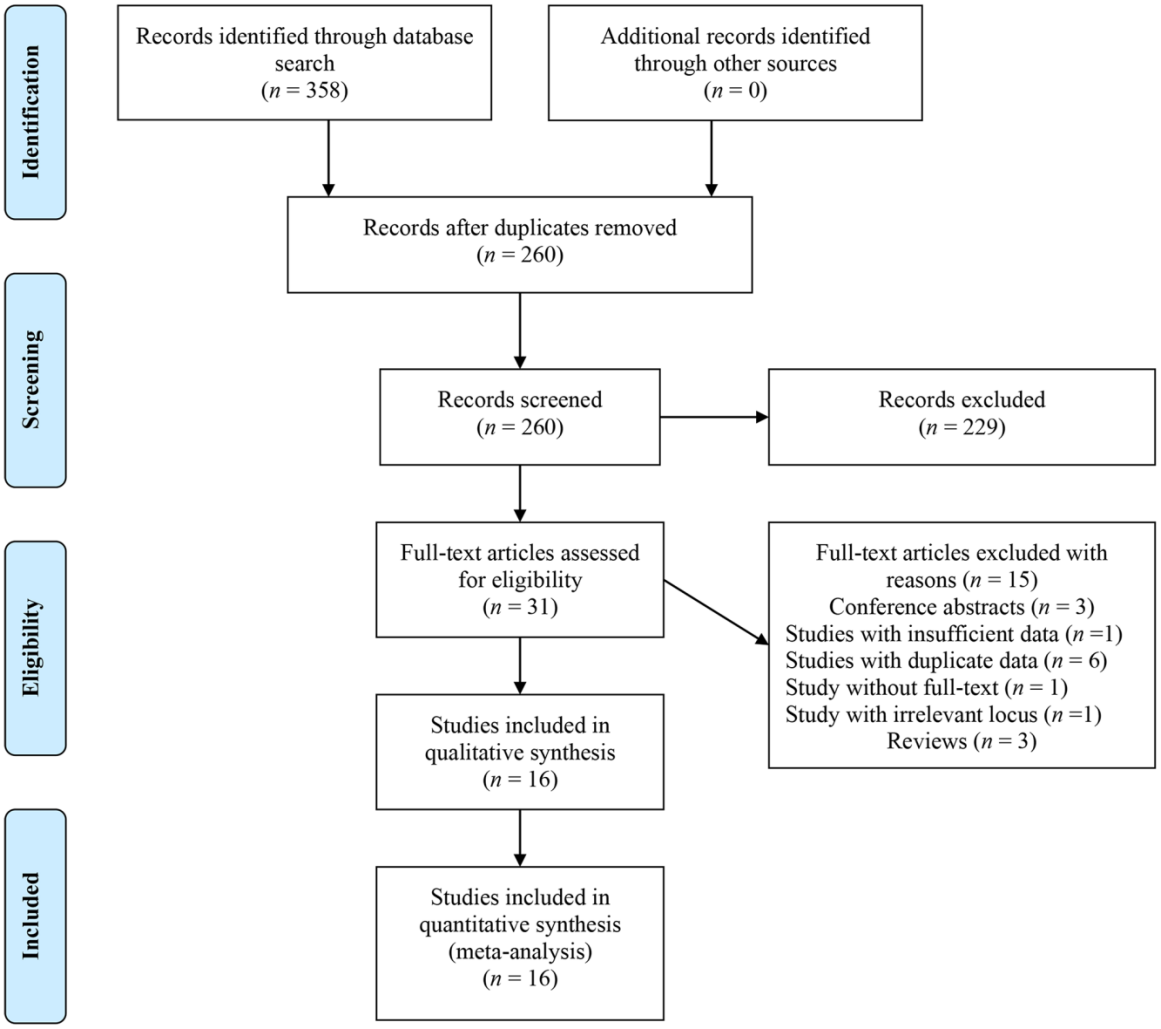
Flow chart of literature identification.

A total of 358 items were initially identified. After removing duplicates and reviewing titles and abstracts, 229 irrelevant articles were excluded, leaving 31 articles for full-text review. Ultimately, 16 articles [10–19, 22–27] were deemed eligible for final data analysis (Figure 1).

### Main characteristics

Table 1 displays the basic characteristics of the included studies. These 16 articles [10–19, 22–27] published from 2008 to 2023 consisted of one cross-sectional study [15], three cohort studies [10, 12, 17], and remaining case-control studies [11, 13, 14, 16, 18, 19, 22–27]. Geographically, five studies [11, 14, 15, 17, 18] were conducted in Asian populations, two [19, 26] in mixed populations, and the rest in European populations. The injury types studied included anterior cruciate ligament injury (ACLI), shoulder dislocation, musculoskeletal injury, tennis elbow, Achilles tendinopathy (TEN), Achilles tendon rupture, tendinopathy, and patellar tendinopathy. Notably, two studies [15, 16] on the *rs1800012* polymorphism and one [18] on the *rs1107946* polymorphism deviated from HWE. All studies scored ≥6 points in quality assessment, indicating high quality (Table 2).

**Table 1:** Main characteristics of included studies.

**Study ID**	**Year**	**Country**	**Ethnicity**	**Gender**	**Study design**	**Genotyping methods**	**Diagnosis**	**Case**			**Control**			**HWE**
								**TT**	**TG**	**GG**	**TT**	**TG**	**GG**	
***Rs1800012***														
Erduran M	2014	Turkey	Caucasian	Both	Case-control	PCR-RFLP	TE	2	32	69	7	35	61	0.53
Ficek K	2013	Poland	Caucasian	Male	Case-control	PCR	ACLI	0	26	65	6	41	96	0.55
Gibbon A	2020	South Africa, UK	Caucasian	Both	Case-control	PCR	TEN, RUP, ACLI	7	119	299	15	125	326	0.49
Haug KBF	2018	Norway	Caucasian	Both	Cohort	TaqMan assay	PTEN	0	14	19	6	23	64	0.07
Khoschnau S	2008	Sweden	Caucasian	Both	Case-control	PCR	CLR, SD	2	99	257	12	83	230	0.20
Le´znicka K	2021	Poland	Caucasian	Both	Case-control	TaqMan assay	MI	8	13	32	9	9	43	< 0.01
Massidda M	2023	Italy	Caucasian	Both	Case-control	PCR-RFLP	ACLI	6	41	38	4	33	44	0.48
Prabhakar S	2019	India	Asian	Both	Case-control	PCR	ACLI	39	7	1	43	7	0	0.60
Shukla M	2020	India	Asian	Both	Case-control	PCR	ACLI	0	14	76	0	12	64	0.46
Sivertsen EA	2019	Norway, Finland	Caucasian	Female	Cohort	TaqMan assay	ACLI	2	38	79	15	205	512	0.29
Stępień-Słodkowska M	2017	Poland	Caucasian	Male	Case-control	TaqMan assay	ACLI	2	46	90	5	39	139	0.28
Zhao D	2020	China	Asian	Both	Cross-sectional	PCR-RFLP	ACLI	1	2	98	1	2	107	< 0.01
***Rs1107946***														
Ficek K	2013	Poland	Caucasian	Male	Case-control	PCR	ACLI	1	30	60	3	33	107	0.81
Gibbon A	2020	South Africa, UK	Caucasian	Both	Case-control	PCR	TEN, RUP, ACLI	5	97	313	12	112	349	0.41
Lopes LR	2023	Brazil	Mixed	Both	Case-control	TaqMan assay	TEND	7	18	29	14	61	110	0.18
Mirghaderi SP	2022	Iran	Asian	Male	Case-control	TaqMan assay	ACLI	33	43	124	29	57	114	< 0.01
Miyamoto-Mikami E	2021	Japan	Asian	Both	Cohort	TaqMan assay	MI	37	87	67	223	652	498	0.70
Perini JA	2022	Brazil	Mixed	Both	Case-control	TaqMan assay	ACLI	10	43	92	15	65	110	0.23
Prabhakar S	2019	India	Asian	Both	Case-control	PCR	ACLI	23	17	7	23	19	8	0.25
Sivertsen EA	2019	Norway, Finland	Caucasian	Female	Cohort	TaqMan assay	ACLI	3	34	82	15	168	549	0.62
Stępień-Słodkowska M	2017	Poland	Caucasian	Male	Case-control	TaqMan assay	ACLI	1	48	89	4	46	133	0.99

Abbreviations: PCR: Polymerase chain reaction; PCR-RFLP: Polymerase chain reaction–restriction fragment length polymorphism; CLR: cruciate ligament rupture; SD: shoulder dislocation; ACLI: anterior cruciate ligament injury; MI: musculoskeletal injury; TE: tennis elbow; TEN: Achilles tendinopathy; TEND: tendinopathy; RUP: Achilles tendon rupture; PTEN: patellar tendinopathy; HWE: Hardy-Weinberg Equilibrium.

**Table 2:** Quality assessment of included studies.

**Study ID**	**Selection**				**Control for important factor**	**Exposure**			
	**Adequate definition of cases**	**Representativeness of cases**	**Selection of control subjects**	**Definition of control subjects**		**Exposure assessment**	**Same method of ascertainment for all subjects**	**Non-response rate**	**Total**
Erduran M, 2014	★	★	☆	★	★★	★	★	★	8
Ficek K, 2013	★	☆	☆	★	★★	★	★	★	7
Gibbon A, 2020	★	☆	☆	★	★☆	★	★	★	6
Haug KBF, 2018	★	☆	☆	★	★☆	★	★	★	6
Khoschnau S, 2008	★	☆	★	★	★☆	★	★	★	7
Le´znicka K, 2021	★	☆	☆	★	★☆	★	★	★	6
Lopes LR, 2023	★	☆	☆	★	★★	★	★	★	7
Massidda M, 2023	★	☆	☆	★	★☆	★	★	★	6
Mirghaderi SP, 2022	★	☆	☆	★	★★	★	★	★	7
Miyamoto-Mikami E, 2021	★	☆	☆	★	★☆	★	★	★	6
Perini JA, 2022	★	☆	☆	★	★★	★	★	★	7
Prabhakar S, 2019	★	☆	☆	★	★☆	★	★	★	6
Shukla M, 2020	★	☆	☆	★	★☆	★	★	★	6
Sivertsen EA, 2019	★	☆	★	★	★☆	★	★	★	7
Stępień-Słodkowska M, 2017	★	☆	☆	★	★☆	★	★	★	6
Zhao D, 2020	★	☆	☆	★	★☆	★	★	★	7

### Meta-analyses and subgroup analyses

#### Association of the rs1800012 polymorphism and MSTIs

**Figure 2:**
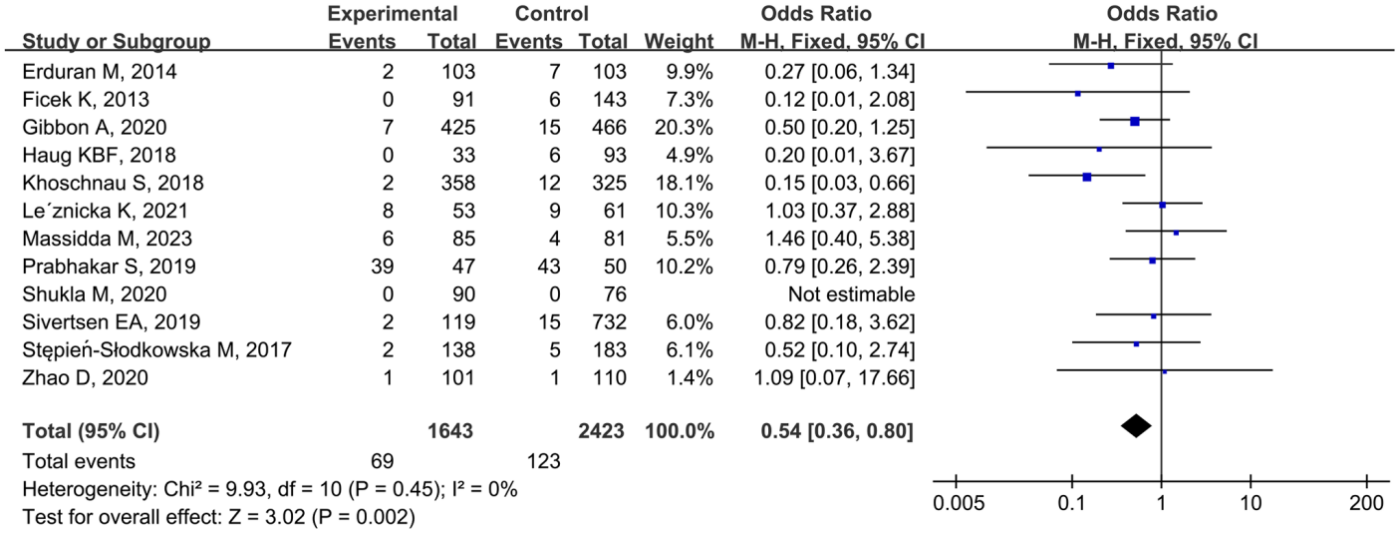
Forest plot of *rs1800012* polymorphism and musculoskeletal soft tissue injuries (TT vs. TG+GG).

Twelve studies [10–16, 22–25, 27] analyzed the correlation between the *rs1800012* polymorphism and MSTIs, involving 1643 cases and 2423 controls. Low heterogeneity was observed, and the fixed-effects model was applied. The combined data showed that TT genotype carriers had a significantly decreased risk of MSTIs (TT vs. GG, OR = 0.53, 95% CI 0.35–0.82, *P* = 0.004; TT vs. TG + GG, OR = 0.54, 95% CI 0.36–0.80, *P* = 0.002; Figure 2).

Stratified analyses by injury type revealed that TG-genotype carriers tended to have an increased risk of ACLI (TG vs. GG, OR = 1.25, 95% CI 1.02–1.55, *P* = 0.03). Ethnicity-based analysis indicated that TT genotype individuals had a reduced risk of MSTIs among Caucasians (TT vs. GG, OR = 0.53, 95% CI 0.34–0.83, *P* = 0.005; TT vs. TG + GG, OR = 0.50, 95% CI 0.32–0.78, *P* = 0.002; Table 3) but not among Asians.

**Table 3:** Association of *COL1A1* gene polymorphisms and musculoskeletal soft tissue injuries.

**Polymorphism/ genetic models**	**Association**			**No. of cohorts**	**Heterogeneity**		**Statistical model**
	**OR**	**95% CI**	***P***		***I^2^* (%)**	***P***	
***Rs1800012***							
Overall							
T vs. G	0.98	0.86–1.12	0.79	12	2	0.43	F
TT vs. GG	0.53	0.35–0.82	0.004	12	9	0.36	F
TG vs. GG	1.16	0.99–1.35	0.07	12	0	0.54	F
TT+TG vs. GG	1.05	0.90–1.22	0.53	12	0	0.56	F
TT vs. TG+GG	0.54	0.36–0.80	0.002	12	0	0.45	F
ACLI							
T vs. G	1.09	0.91–1.30	0.34	8	0	0.61	F
TT vs. GG	0.66	0.34–1.26	0.20	8	0	0.63	F
TG vs. GG	1.25	1.02–1.55	0.03	8	0	0.72	F
TT+TG vs. GG	1.19	0.97–1.46	0.10	8	0	0.65	F
TT vs. TG+GG	0.88	0.73–1.06	0.18	8	0	0.64	F
Caucasian							
T vs. G	0.99	0.87–1.13	0.84	9	26	0.21	F
TT vs. GG	0.53	0.34–0.83	0.005	9	25	0.22	F
TG vs. GG	1.17	1.00–1.36	0.05	9	13	0.33	F
TT+TG vs. GG	1.05	0.91–1.23	0.50	9	12	0.33	F
TT vs. TG+GG	0.50	0.32–0.78	0.002	9	15	0.31	F
Asian							
T vs. G	0.90	0.51–1.61	0.73	3	0	0.85	F
TT vs. GG	0.60	0.08–4.58	0.63	3	0	0.55	F
TG vs. GG	0.94	0.44–1.98	0.86	3	0	0.82	F
TT+TG vs. GG	0.93	0.45–1.92	0.85	3	0	0.78	F
TT vs. TG+GG	0.83	0.30–2.31	0.72	3	0	0.84	F
***Rs1107946***							
Overall							
T vs. G	1.05	0.94–1.18	0.40	9	0	0.45	F
TT vs. GG	1.05	0.80–1.37	0.73	9	0	0.67	F
TG vs. GG	1.05	0.90–1.22	0.57	9	26	0.22	F
TT+TG vs. GG	1.05	0.91–1.21	0.51	9	13	0.33	F
TT vs. TG+GG	1.09	0.85–1.40	0.50	9	0	0.65	F
ACLI							
T vs. G	1.04	0.89–1.21	0.61	7	0	0.45	F
TT vs. GG	0.92	0.63–1.37	0.69	7	0	0.88	F
TG vs. GG	1.07	0.89–1.30	0.47	7	42	0.11	F
TT+TG vs. GG	1.06	0.88–1.27	0.53	7	28	0.21	F
TT vs. TG+GG	0.99	0.69–1.42	0.96	7	0	0.83	F
Caucasian							
T vs. G	1.09	0.91–1.31	0.36	4	31	0.22	F
TT vs. GG	0.63	0.30–1.30	0.21	4	0	0.59	F
TG vs. GG	1.22	0.99–1.50	0.07	4	30	0.23	F
TT+TG vs. GG	1.17	0.95–1.43	0.14	4	35	0.20	F
TT vs. TG+GG	0.60	0.29–1.24	0.17	4	0	0.62	F
Asian							
T vs. G	1.04	0.88–1.23	0.65	3	0	0.69	F
TT vs. GG	1.16	0.83–1.61	0.38	3	0	0.90	F
TG vs. GG	0.88	0.68–1.15	0.37	3	0	0.47	F
TT+TG vs. GG	0.96	0.75–1.22	0.74	3	0	0.59	F
TT vs. TG+GG	1.20	0.89–1.61	0.22	3	0	0.97	F

Abbreviations: OR: odds ratios; CI: confidence interval; F: fixed-effects model; R: random-effects model; ACLI: anterior cruciate ligament injury.

#### Association of the rs1107946 polymorphism and MSTIs

**Figure 3:**
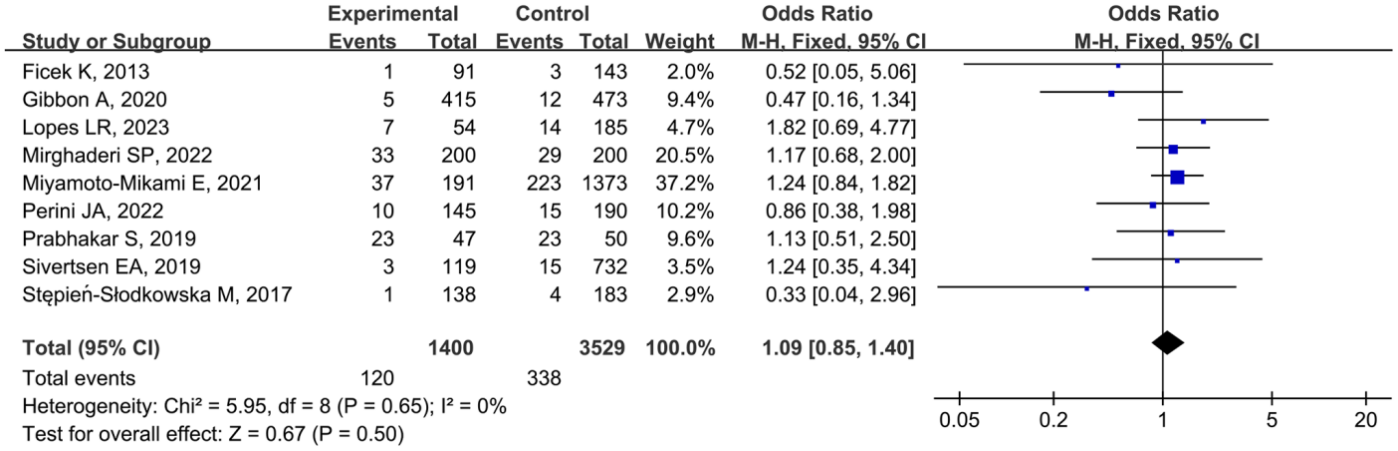
Forest plot of *rs1107946* polymorphism and musculoskeletal soft tissue injuries (TT vs. TG+GG).

Nine studies [11–13, 17–19, 23, 25, 26] examined the association of the *rs1107946* polymorphism with MSTIs, involving 1400 cases and 3529 controls. As minimal between-study heterogeneity was detected, the fixed-effects model was used. The pooled data did not show any significant association between the *rs1107946* polymorphism and MSTIs (TT vs. TG + GG, OR = 1.09, 95% CI 0.85–1.40, *P* = 0.50; Figure 3). Subgroup analyses by injury type and ethnicity also revealed no significant association (Table 3).

### Sensitivity analysis and publication bias

**Figure 4:**
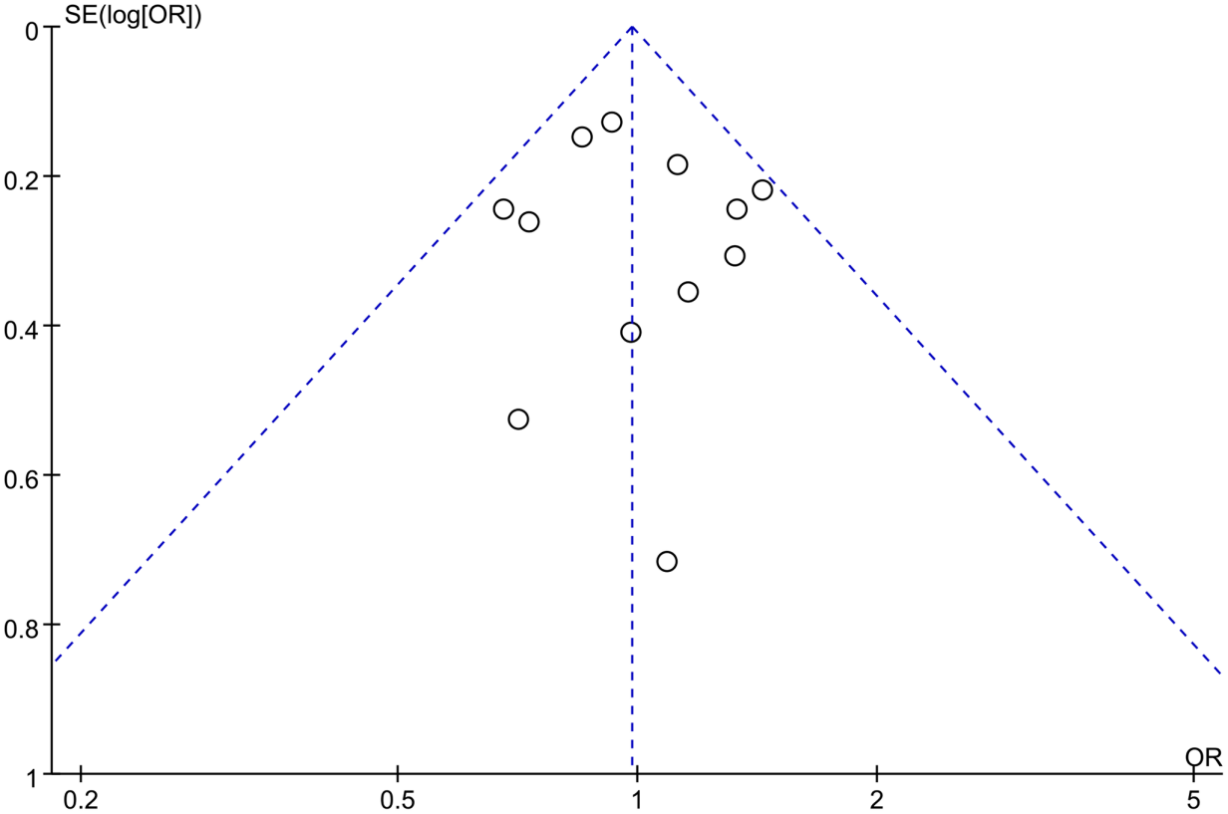
Funnel plot of *rs1800012* polymorphism and musculoskeletal soft tissue injuries (TT vs. TG+GG).

Sequential removal of each study did not result in significant fluctuations in the re-pooled ORs and 95% CIs, indicating the stability and robustness of the results. Funnel plots displayed symmetrical patterns, suggesting no significant publication bias (Figure 4). Egger’s and Begg’s linear regression tests did not find significant publication bias (Supplementary Table 1).

### Trial sequential analysis

**Figure 5:**
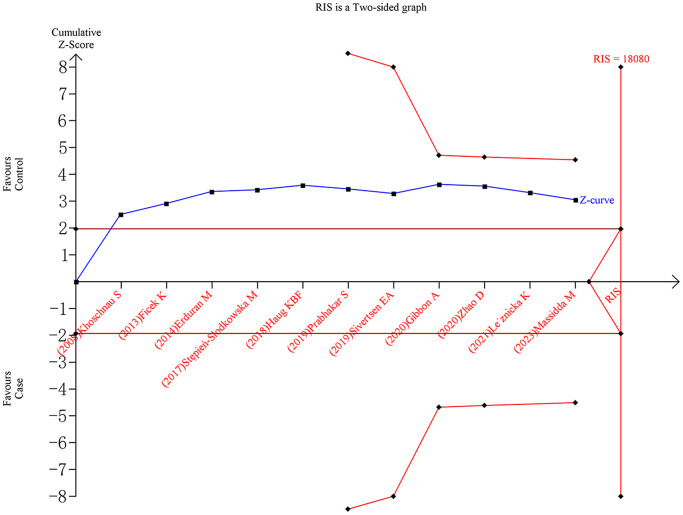
Trial sequential analysis of *rs1800012* polymorphism and musculoskeletal soft tissue injuries (TT vs. TG+GG).

For the *rs1800012* polymorphism, the cumulative Z-curves did not surpass the TSA monitoring boundaries or the RIS line under five genetic models, indicating insufficient evidence and the need for further studies (Figure 5). In contrast, for the *rs1107946* polymorphism, the cumulative Z-curves exceeded the RIS line but did not surpass the TSA monitoring boundaries under the allele, heterozygote, and dominant models, suggesting sufficient evidence. However, the cumulative Z-curves did not cross the TSA monitoring boundaries or the RIS line under the homozygote and recessive models, indicating the need for additional studies to obtain definitive outcomes.

## DISCUSSION

Understanding the etiology of MSTIs is crucial for risk reduction and improved prognosis. However, the causative mechanisms of MSTIs are complex and not fully understood. Genetic factors play a significant role in MSTIs, as indicated by mounting evidence and the postulation of familial predisposition by researchers [28, 29]. Over 80 loci have been associated with MSTIs [30]. The association of *COL1A1* polymorphisms with MSTIs has been extensively studied but with inconsistent findings. Therefore, this meta-analysis was conducted to synthesize existing evidence and clarify this association. Our findings support a protective role of the TT genotype of the *rs1800012* polymorphism in MSTIs, particularly among Caucasians. However, the *rs1107946* polymorphism showed no association with MSTIs across different ethnicities and injury types.

Tendons and ligaments are vital components of the musculoskeletal system, with COL1 playing a crucial role. COL1, also known as fibril-forming collagen, is abundant and widely used in tissue engineering due to its structural properties. Although COL1 fibers have high tensile strength and are resistant to most proteases, abnormal accumulation can lead to fibrotic diseases. COL1 is encoded by the *COL1A1* and *COL1A2* genes, which are respectively translated into α1 and α2 polypeptide chains in a coordinated pattern. Two α1 chains interplay with one α2 to form a triple-helical structure. The genetic aspects of the synthesis, function, and degradation of COL1 fibers and its association with diseases are under active studies [31]. In human genome, the *COL1A1* gene is mapped to chromosome 17q21.33, which is 18 kb in length and consists of 52 exons.

*COL1A1* gene variants are associated with various musculoskeletal disorders, including intervertebral disc degeneration [32], osteoporosis [33], osteoarthritis [34], and osteogenesis imperfecta [35]. The *rs1800012* polymorphism is a guanine (G) to thymine (T) transversion within an Sp1 binding element in intron 1 (position +1245) of the *COL1A1* gene [36]. Mann et al. [37] found that the T allele of *rs1800012* is associated with stronger transcriptional activity and higher α1 to α2 chain ratio, which was reflected by the increased mRNA ratio of *COL1A1* to *COL1A2*. Ireland et al. [38] observed elevated levels of *COL1* and *COL3* mRNAs in samples from individuals with TEN.

*Rs1107946* is a frequently studied variant located in the proximal promoter of the *COL1A1* gene, specifically at position-1997 in intron 1. In contrast to the *rs1800012* polymorphism, the G allele of *rs1107946* exhibits higher transcriptional activity than the T allele. Individuals with the TT genotype for the *rs1107946* polymorphism have a significantly lower proportion of α1 chain homotrimers, potentially leading to increased resistance against injury [17]. Furthermore, *rs1107946* is strongly linked with the *rs1800012* polymorphism [39]. Ficek et al. [23] reported that while the *rs1800012* and *rs1107946* polymorphisms were not directly associated with ACLI, the *COL1A1* G-T haplotype demonstrated a protective effect against ACLI among professional soccer players [23]. Additionally, Jin et al. [40] observed that *COLIA1* variants regulate transcription through DNA-protein interactions. They suggested that the G-del-T haplotype (*rs1107946-rs2412298*-*rs1800012*) leads to higher transcriptional activity of *COL1A1*, resulting in increased production of the α1 chain, disruption of the α1 and α2 chain ratio, and generation of mature COL1 with an altered structure. Perini et al. [19] found that a separate polymorphism in the *COL1A1* and *COL1A2* genes was not directly associated with ACLI, but the cumulative effects of *COL1A1* and *COL1A2* variants might contribute to the risk of ACLI among athletes.

Wang et al. [41] conducted a meta-analysis involving 933 cases and 1381 controls to investigate the relationship between *rs1800012* and tendon-ligament injuries. Compared with Wang et al.’s study, the present study has several advantages. First, new evidence was incorporated into this study, leading to an increase in the sample size and enhancement of statistical power. Moreover, while Wang et al.’s study focused solely on Caucasian participants, our study included subjects from Asian and Brazilian populations. Ethnicity stratification was performed to assess potential differences among variant ethnicities. Furthermore, the *rs1107946* polymorphism, a crucial locus within the *COL1A1* gene, was examined in this meta-analysis. Additionally, TSA was conducted to evaluate the adequacy of the current data to achieve positive outcomes.

**Figure 6:**
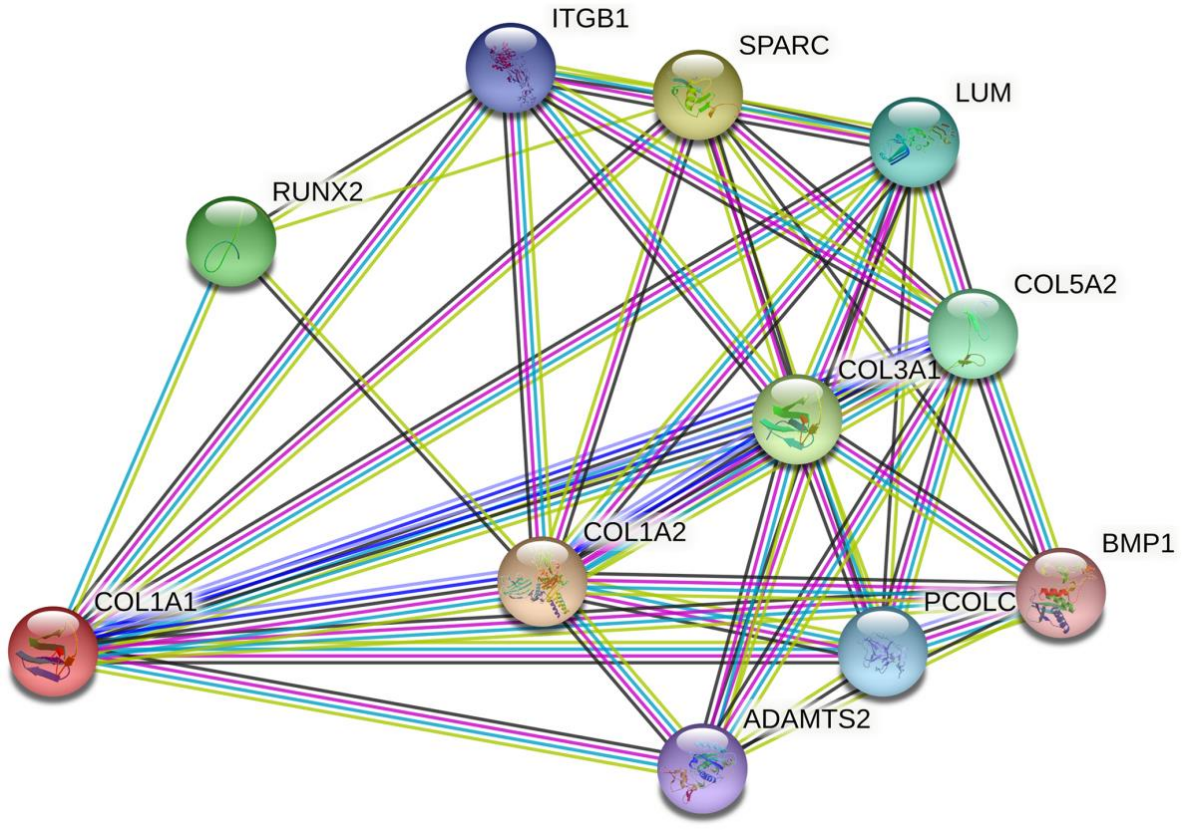
Network of COL1A1 with its potentially functional partners.

Several limitations should be acknowledged in this study. First, most of the included studies had small sample sizes, which may have limited statistical power, as also indicated by TSA results. Second, the majority of research focused on Caucasians and Asians, necessitating the replication of findings in other ancestral groups to ensure generalizability. Third, all studies were observational, indicating a lower level of evidence. Fourth, due to limited data availability, adjustments for confounding factors such as gender, age, body mass index, occupation, and exercise intensity were not feasible. Fifth, the participant group in this study was heterogeneous, encompassing various injuries and occupations. Lastly, the meta-analysis only analyzed two loci within the *COL1A1* gene, whereas MSTIs are complex and likely influenced by multiple genes interacting synergistically. The interaction network of *COL1A1* and its related genes, including *ADAMTS2*, *COL1A2*, *COL5A2*, and *RUNX2*, is illustrated in Figure 6, suggesting a potential role of *COL1A1* in the pathogenesis of MSTIs.

In conclusion, this study supports the protective effect of the TT genotype of the *rs1800012* polymorphism against MSTIs, particularly among Caucasians. However, the *rs1107946* polymorphism does not show an association with MSTIs. Given the limitations outlined, larger-scale prospective studies across diverse ethnic backgrounds are warranted to validate these findings and provide a more comprehensive understanding of genetic influences on MSTIs.

## Supplementary Materials

Supplementary Table 1
