# Supplementary material for: Associations of collagen type 1 α1 gene polymorphisms and musculoskeletal soft tissue injuries: a meta-analysis with trial sequential analysis
Source: Aging (Albany NY). 2024 May 22;16(10):8866–79. doi: 10.18632/aging.205846 (PMC11164502; doi:10.18632/aging.205846)
Supplement: Supplementary Table 1 [file aging-16-205846-s001.pdf]

SUPPLEMENTARY TABLE

Supplementary Table 1. Results of Egger's and Begg's tests.

Polymorphisms	Egger's test		Begg's test	
	t-value	*P*-value	Z-value	*P*-value
Rs1800012				
T vs. G	0.61	0.055	0.21	0.837
TT vs. GG	−2.21	0.557	1.56	0.119
TG vs. GG	0.87	0.407	0.89	0.373
TT+TG vs. GG	1.54	0.155	0.89	0.373
TT vs. TG+GG	−2.32	0.051	1.56	0.119
Rs1107946				
T vs. G	0.90	0.397	0.73	0.466
TT vs. GG	−1.24	0.253	0.94	0.348
TG vs. GG	0.78	0.459	1.15	0.251
TT+TG vs. GG	1.09	0.311	1.15	0.251
TT vs. TG+GG	−1.76	0.122	1.15	0.251
